# Mental health front-liners: Police officers’ knowledge and attitudes towards suicide in Malta

**DOI:** 10.1192/j.eurpsy.2024.518

**Published:** 2024-08-27

**Authors:** D. Zammit, M. Bezzina Xuereb

**Affiliations:** Mental Health Services, Mount Carmel Hospital, Attard, Malta

## Abstract

**Introduction:**

Police-officers are in a strategic position of providing the first immediate response to a crisis as mental health frontliners.

**Objectives:**

In this nation-wide cross-sectional study, we explored knowledge and attitudes towards suicide in the local police force, a crucial first step in the design and implementation of effective suicide prevention programmes.

**Methods:**

An online, anonymous questionnaire was distributed to all local police-officers (n=2600). It contained questions about their demographics and their experience with suicide while on duty, along with 34 statements from the validated tool Attitudes Towards Suicide (ATTS) (Renberg & Jacobsson. Suicide Life Threat Behav. 2003; 33 52-64), scored on a 5-point Likert Scale (1 = Strongly Disagree, 5 = Strongly Agree).

**Results:**

The sub-scale “*Suicide as a right*” was positively correlated with “*Tabooing*” (r (201) = .25, p=<.001), “*Normal-common *” (r (201) = .29, p=<.001), and “*Resignation*” (r (201) = .47, p=<.001), but negatively correlated with “*Incomprehensibility*” (r (201) = -.26, p=<.001), and “*Preparedness to Prevent*” (r (201) = -.19, p=<.001), meaning such individuals had a more permissive attitude towards suicide. On the other hand, the subscale *Preventability* was found to be positively correlated with *Incomprehensibility* (r (201) = .21, p=<.001) and *Preparedness to Prevent* (r (201) = .30, p=<.001).

Females scored higher in the sub-scale *Non-communication* (M=3.40, 95% CI [3.29, 3.51]) while males scored higher in *Preventability* (M=3.35, 95% CI [3.27, 3.44]). The higher the educational status of police-officers, the more they adopt a pro-prevention attitude to suicide (M=3.67, 95% CI [3.44, 3.89]) and the more likely they are to appreciate that suicidal thoughts and behaviour can be common (M=3.40, 95% CI [3.20, 3.60]). Participants with a mixed/different composition at home (M=4.05, 95% CI [3.86, 4.24]) and/or have experienced only between 0 to 2 situations related to suicide in the past one year alone (M=4.05, 95% CI [3.94, 4.16]), were the most likely to feel prepared to prevent suicide.

**Conclusions:**

This study brings out different attitudes police-officers hold towards different aspects of suicide, influenced by their gender, educational background, personal life at home and total exposure to suicide during their career. Training programmes can help improve their knowledge and attitudes towards suicide, leading to a more positive behavioural response to individuals in crisis and create a safer environment. Malta, through an EU-funded programme, is currently investing its resources on drafting a national suicide prevention strategy, and such educational opportunities for our frontliners will ensure we have the right tools in screening, identifying, treating, and saving more lives.

Abbreviations: *M: Mean score; CI: Confidence Interval*

**Disclosure of Interest:**

None Declared

